# *Mahuang Fuzi Xixin* Decoction Attenuates Th1 and Th2 Responses in the Treatment of Ovalbumin-Induced Allergic Inflammation in a Rat Model of Allergic Rhinitis

**DOI:** 10.1155/2017/8254324

**Published:** 2017-07-13

**Authors:** Mengyue Ren, Qingfa Tang, Feilong Chen, Xuefeng Xing, Yao Huang, Xiaomei Tan

**Affiliations:** ^1^School of Traditional Chinese Medicine, Southern Medical University, Guangzhou 510515, China; ^2^Guangdong Provincial Key Laboratory of Chinese Medicine Pharmaceutics, Southern Medical University, Guangzhou 510515, China

## Abstract

Allergic rhinitis (AR) is one of the most common allergic diseases, which adversely affect patients' quality of life. *Mahuang Fuzi Xixin* decoction (MFXD) has been widely used to treat AR in clinics in Asian countries. This study investigated the effect and possible therapeutic mechanisms of MFXD in the treatment of AR. A Wistar rat model of ovalbumin- (OVA-) induced AR was established and then treated with three doses of MFXD; AR symptoms, serum total immunoglobulin E, histamine, histopathological features, and release and expression of factors related to type 1 helper T (Th1) and type 2 helper T (Th2) responses were analyzed. Our study demonstrated that MFXD has a good therapeutic effect on OVA-induced allergic inflammation in an AR rat model as manifested in reduced frequencies of sneezing and nasal scratching and in reduced serum levels of total IgE and HIS. In addition, MFXD regulates imbalance in Th1/Th2 cells caused by AR by simultaneously attenuating Th1 and Th2 responses, such as by reducing the serum levels of IFN-*γ* and IL-4 and mRNA expression levels of IFN-*γ*, IL-4, GATA-3, and STAT-6. This study provided valuable information on the immunoregulatory effect of MFXD for the treatment of AR in future clinical studies.

## 1. Introduction

Allergic rhinitis (AR) is a type I allergic disease induced by an immunoglobulin E- (IgE-) mediated inflammation and characterized by paroxysmal nasal obstruction, rhinorrhea, nasal itching, and sneezing [1, 2]. As an extremely common disease, AR has affected more than 500 million people worldwide over the last 20 years [3]. AR is not a severe disease, but it significantly impacts patients' quality of life, school performance, and work productivity and is considered an economic burden; moreover, AR has multiple comorbidities, such as asthma, conjunctivitis, headache, nasal polyps, sinusitis, and otitis media [3, 4].

The allergic sensitization procedure for AR was well established in the 1970s. When persistently exposed to certain concentrations of allergens, an antigen-presenting cell presents the allergens to CD4^+^ T lymphocytes, which in turn release cytokines that stimulate B lymphocytes to differentiate into plasma cells; as a result, production of immunoglobulin E (IgE) is promoted. Individuals become sensitized when IgE antibodies bind to receptors on mast cells and eosinophils; when they are exposed to allergens once again, IgE-mediated inflammation is stimulated, resulting in AR symptoms [5–7].

The imbalance in type 1 helper T (Th1) cells and type 2 helper T (Th2) cells has been considered the main induction factor in IgE-mediated allergic inflammation [6, 8–11]. When infected with AR, the differentiated proportion into Th2 cells will increase significantly and interleukin-4 (IL-4) (mainly released by Th2 cells) secretion prominently increased to accelerate the production of IgE and simultaneously inhibit Th1 response such as the release of interferon-*γ* (IFN-*γ*), which is called the imbalance of Th1/Th2 [9, 12–14]. However, researchers have questioned the Th1/Th2 imbalance theory and the weakened immunological drive in the Th1 direction that leads to AR; Randolph et al. found that the Th1 response plays a dominant role in the early phase of ovalbumin- (OVA-) induced mouse airway inflammation [15].

The most popular medications currently used for AR are oral H1 antihistamines and intranasal corticosteroids, which are constantly combined with immunotherapy; these medications can control this allergic disease within either short or long term [5, 16]. However, a series of side effects, such as mild drowsiness, deep sleep, dizziness, lassitude, inability to concentrate, and arrhythmia, may occur during treatment [3]. Given these side effects, many patients have used complementary therapies, such as Chinese herbal medicine and acupuncture for the treatment of AR, and these therapies are used due to their few side effects and low toxicity [17–19].

*Mahuang Fuzi Xixin* decoction (MFXD) is an extract of a classical Chinese traditional formula consisting of *Ephedrae* (*Mahuang* in Chinese, dried herbaceous stems of *Ephedra sinica* Stapf), *Radix Aconiti Lateralis* (*Fuzi* in Chinese, dried lateral roots of *Aconitum carmichaelii* Debx), and *Asarum* (*Xixin* in Chinese, dried roots and rhizomes of *Asarum sieboldii* Miq.) at a dry weight ratio of 2 : 3 : 1; MFXD is used to treat common cold, migraine, asthma, rheumatoid arthritis, and AR [20, 21]. *Ephedrae* has been widely used in China to treat asthma and common cold, and alkaloids such as ephedrine and pseudoephedrine are its main effective constituents [22, 23]. Similarly, *Aconitum* alkaloids, especially lowly toxic monoester alkaloids, such as benzoylaconine, benzoylhypaconine, and benzoylmesaconine, have been identified as the main pharmacologic components of *Radix Aconiti Lateralis*, making it as an effective treatment against rheumatoid arthritis and asthma [24, 25]. *Asarum* has been generally used to treat common cold, migraine, and bronchitis, and its main effective components are the essential oils methyleugenol, *α*-pinene, and safrole [26, 27]. MFXD is a traditional medicine used in China, Japan, and other Asian countries to treat AR; we previously demonstrated that MFXD is an effective treatment for allergic inflammation in a guinea pig model of AR [28]. However, the therapeutic mechanism of MFXD against allergic inflammation remains unclear.

In this study, a rat model of OVA-induced AR was used to investigate the effect of MFXD on allergic inflammation and Th1 and Th2 immune responses associated with AR were further examined to elucidate the possible therapeutic mechanisms of MFXD.

## 2. Materials and Methods

### 2.1. Animal

Specific pathogen-free (SPF) adult male Wistar rats (180 ± 20 g) were obtained from the Experimental Animal Center of Southern Medical University (number 44002100009161), and this study was approved by the Institutional Animal Care and Use Committee of Southern Medical University, Guangzhou, China (Approval number L2016072). Rats were housed in the SPF Experimental Animal Center of Southern Medical University with a relative humidity of 40–70% and at a temperature of 20–24°C under lighting controls (12 h light/dark cycle). All rats had free access to standard food and water and were allowed to be acclimated for seven days before the experiment.

### 2.2. Preparation of Herb Extract

The preparation of MFXD was conducted as described in Treatise on Febrile Diseases, an ancient Chinese medical book. *Ephedrae* (60 g; Guangzhou Zhixin Chinese Medicine YinPian Co. Ltd., Guangzhou, China) was immersed in water (2700 mL) for 30 min and boiled for 20 min. *Radix Aconiti Lateralis* (90 g; Guangzhou Zhixin Chinese Medicine YinPian Co. Ltd., Guangzhou, China) and *Asarum* (30 g; Kangmei Pharmaceuticals Co. Ltd., Puning, China) were subsequently added and then simmered for another 90 min. Filtered water extract was concentrated to 1.52 g·mL^−1^ under reduced pressure and then reconstituted in distilled water to achieve the required dose for all subsequent experiments.

### 2.3. Fingerprint Analysis of MFXD through Ultra Performance Liquid Chromatography-Tandem Mass Spectrometry (UPLC-MS/MS)

UPLC-MS/MS was used to analyze the chemical composition of MFXD. Chromatographic analysis was performed on an Agilent 1290 Infinity LC system (Agilent Technologies, Wilmington, Delaware, USA) and on a 6410B Triple Quadrupole Mass Spectrometer (Agilent Technologies, USA). In brief, the analytes of MFXD were separated on a Zorbax SB-Aq column (100 mm × 2.1 mm, 3.5 *μ*m; Agilent Technologies, USA) with a mobile phase consisting of acetonitrile (A) and 0.1% aqueous solution of formic acid (B); the following gradient program was used: 0% A at 0–2 min, 0% A–5% A at 2–5 min, 5% A at 5–8 min, 5% A–20% A at 8–15 min, 20% A–35% A at 15–28 min, 35% A–50% A at 28–31 min, 50% A–55% A at 31–36 min, 55% A–95% A at 36–45 min, and 95% A–100% A at 45–55 min. The injection volume was 1 *μ*L, the flow rate is 0.4 mL/min, and the column temperature was 25°C.

### 2.4. Preparation of AR Rat Models and Drug Administration

**Figure 1:**
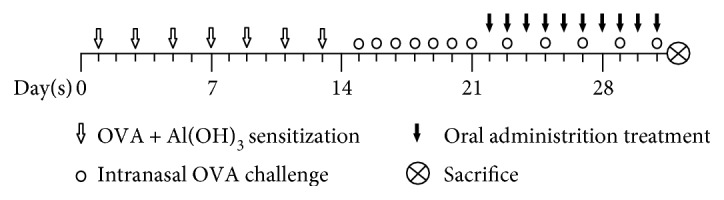
Schematic diagram of preparation of AR model rats and MFXD administration. Rats were intraperitoneally sensitized with OVA and Al(OH)_3_ dissolved in physiological saline once every other day for 2 weeks and then challenged through nasal instillation with 5% OVA into each nasal cavity once daily from day 15 to day 21. Rats in MFXD, loratadine, and model groups were orally administered with three doses of MFXD, loratadine solution, and water once daily from day 22 to day 31, respectively, and were nasally instilled with OVA solution once every other day to maintain the nasal stimulation. Rats in the control group were sensitized with Al(OH)_3_, challenged with saline, and orally administered with water. All the rats were sacrificed on day 31.

AR rat models were established as described previously [29] with minor modifications, as summarized in Figure 1. In brief, the rats were intraperitoneally sensitized with 0.3 mg of OVA (albumin egg, Sigma, MO, USA) and 30 mg of Al(OH)_3_ (Damao Chemical Reagent Factory, China) dissolved in 1 mL of physiological saline once every other day for 2 weeks. The rats were subsequently challenged through nasal instillation with 50 *μ*L of OVA solution (5%, dissolved in physiological saline) into each nasal cavity once daily from day 15 to day 21. Forty AR rats were randomly divided into five groups (eight rats per group), namely, AR model, MFXD (1.9 g/kg), MFXD (3.8 g/kg), MFXD (7.6 g/kg), and loratadine (1 mg/kg, Aobang Pharmaceuticals Co. Ltd., Sichuan, China) groups. Rats in three MFXD groups were orally administered daily with three doses MFXD 1 h before nasal challenge for 10 days (day 22 to day 31) according to our preliminary study; rats in the loratadine and AR model groups were simultaneously treated intragastrically with loratadine solution (1 mg/kg) and filtered water, respectively. The rats in the control group were sensitized with Al(OH)_3_, challenged with saline, and orally administered with filtered water synchronously. During the period of oral administration, all of the AR rats were challenged through nasal instillation with 50 *μ*L of 5% OVA solution once every other day to maintain the nasal stimulation.

### 2.5. Evaluation of Nasal Symptoms

On the last day (day 31), the rats were placed in observation cages for approximately 10 min for acclimatization after oral administration. The frequencies of sneezing and nasal scratching in the rats were counted for 30 min immediately after the last challenge involving nasal instillation with 50 *μ*L of 5% OVA solution. After the evaluation of nasal symptoms, blood samples were collected from rats by using the abdominal aortic method under anesthesia; sera were obtained through centrifugation (3000 rpm) for 10 min and then stored at −80°C. Nasal mucosa samples were removed for further histopathological and qRT-PCR analyses.

### 2.6. Histopathological Examination

Nasal mucosa samples were fixed in 4% paraformaldehyde (Biosharp, Hefei, Anhui, China) for 24 h and then embedded in paraffin. Paraffin-embedded tissue samples were cut into 4 *μ*m thick sections and stained with hematoxylin and eosin (HE) and toluidine blue (TB, for mast cells). Histopathological changes were evaluated and photographed using an orthorhombic optical photomicroscope (Eclipse Ci, Nikon, Japan).

### 2.7. Detection of Total IgE, HIS, IFN-*γ* (Th1 Cytokine), and IL-4 (Th2 Cytokine) in Rat Serum

Serum levels of total IgE and HIS and those of IFN-*γ* and IL-4 were measured via enzyme-linked immunosorbent assay (ELISA) according to the manuals of rat HIS and IgE Elisa Assay kits (Nanjing Jiancheng Bioengineering Institute, Nanjing, China) and rat IFN-*γ* and IL-4 ELISA kits (CUSABIO, Wuhan, China), respectively. The minimum detection limits of IgE, HIS, IFN-*γ*, and IL-4 were 0.05 U/mL, 0.5 ng/mL, 0.625 pg/mL, and 1.56 pg/mL, respectively.

### 2.8. Detection of CD3^+^CD4^+^IFN-*γ*^+^ Th1 and CD3^+^CD4^+^IL-4^+^ Th2 Cells through Flow Cytometry

Peripheral blood mononuclear cells (PBMCs) of rats were separated from the blood sample according to the instruction provided in Rat Peripheral Blood Lymphocyte Separation Kit (Solarbio, Beijing, China) and placed in a tube containing RPMI 1640 (Gibco, CA, USA). The PBMCs were stimulated with 81 ng/mL phorbol 12-myristate 13-acetate (eBioscience, CA, USA), 1.34 *μ*g/mL ionomycin (eBioscience, CA, USA), and 3.0 *μ*g/mL Brefeldin A (BFA) (eBioscience, CA, USA) for 6 h in 5% CO_2_ humidified incubator (Thermo Fisher Scientific, Shanghai, China). Cells were subsequently surface-stained with fluorescein isothiocyanate- (FITC-) labeled anti-rat CD3 and allophycocyanin- (APC-) labeled anti-rat CD4 antibodies (BD Biosciences, CA, USA) at room temperature for 30 min in the dark and then fixed and permeabilized with intracellular fixation and permeabilization buffer (eBioscience, CA, USA), respectively, according to the manufacturer's instruction. After being washed in phosphate-buffered saline (PBS) and after centrifugation, the cells were divided equally and then incubated with phycoerythrin- (PE-) labeled anti-rat IFN-*γ*, PE-labeled anti-rat IL-4, and PE-labeled anti-rat lgG1 antibodies (BD Biosciences, CA, USA) at room temperature for 15 min in the dark. The stained cells were washed once and detected by a FACSCalibur flow cytometer (BD Biosciences, San Jose, CA, USA), and the results were analyzed with CellQuest software (BD FACSDiva, USA).

### 2.9. RNA Extraction and Quantitative Real-Time PCR (qRT-PCR) Analysis of the Nasal Mucosa

Total RNA was isolated from the nasal mucosa by using a Total RNA Extraction Kit (Solarbio, Beijing, China). Complementary DNA (cDNA) was synthesized through reverse transcription reaction of the extracted RNA by using a Bestar™ qPCR RT Kit (DBI^®^ Bioscience, Shanghai, China) according to the manufacturer's instruction under the following temperature conditions: 37°C for 15 min and 98°C for 5 min. qPCR was performed on an Applied Biosystems 7500 Real-Time PCR System (Life Technologies, USA) by using 10 *μ*L of qPCR MasterMix, 0.5 *μ*L of forward primer (10 *μ*M), 0.5 *μ*L of reverse primer (10 *μ*M), and 1 *μ*L of cDNA using Bestar^®^ SYBR Green qPCR MasterMix Reagent (DBI Bioscience, Shanghai, China). The forward primer and reverse primer sequences of IFN-*γ*, IL-4, T-bet, GATA-3, STAT-1, STAT-6, and *β*-actin (internal reference) are listed in Table 1. The conditions for PCR were as follows: initial denaturation at 95°C for 5 min and then denaturation at 95°C for 10 s, annealing at 55°C for 30 s, and extension at 72°C for 20 s for 40 cycles. The mRNA levels of the six target genes were normalized relative to *β*-actin by using cycle threshold (Ct) values.

### 2.10. Statistical Analysis

The results of the four experiments were analyzed using SPSS for Windows version 16.0 (SPSS, Chicago, IL, USA) and expressed as mean ± standard deviation (SD). Between-groups comparisons were performed using one-way ANOVA and analyzed by LSD *t*-test (when equal variances are assumed) or Tamhane's T2 test (nonparametric test; when equal variances are not assumed), and *p* < 0.05 indicated statistical significance.

## 3. Results

### 3.1. MFXD Fingerprint

**Figure 2:**
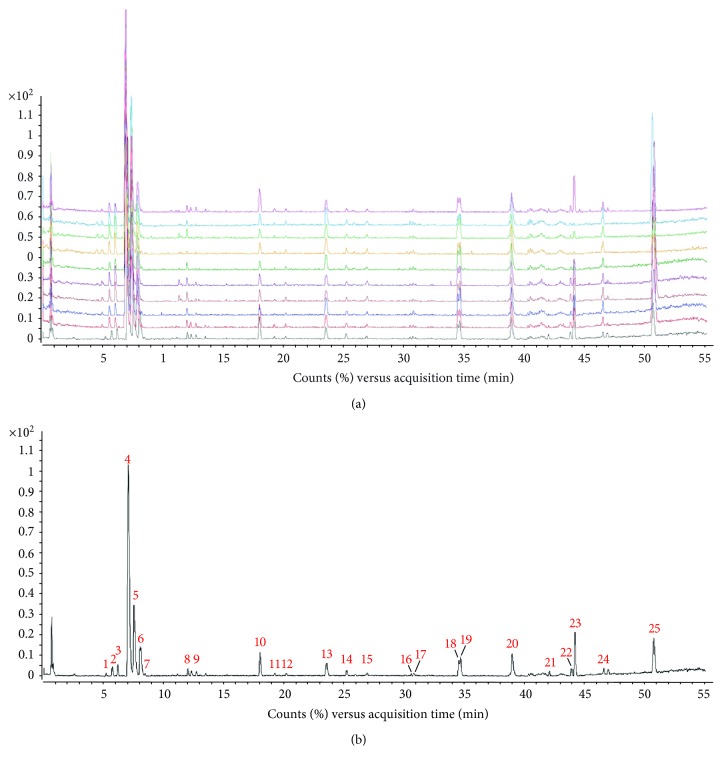
Total ion chromatograms of MFXD. 10 groups of MFXD consisting of different batches of *Mahuang*, *Fuzi*, and *Xixin* were diluted to 0.06 g/mL and precipitated with equal methanol. The supernatants were obtained by centrifugation (10000 rpm) for 10 min and analyzed by UPLC-MS/MS using a mobile phase consisting of acetonitrile (a) and 0.1% formic acid aqueous solution (b) with a gradient program. The injection volume was 1 *μ*L, the flow rate is 0.4 mL/min, and the column temperature was 25°C. (a) The total ion chromatograms of 10 groups of MFXD. (b) The MFXD fingerprint.

The components of 10 groups of MFXD consisting of different batches of *Mahuang*, *Fuzi*, and *Xixin* were analyzed by UPLC-MS/MS.  Figure 2(a) shows the total ion chromatograms of the 10 groups of MFXD, which displayed a high degree of similarity. As shown in Figure 2(b), 25 peaks in the total ion chromatograms of MFXD were assigned as common peaks, and the relative standard deviations of the relative retention time (RRT) of these 25 common peaks were lower than 1.0%, indicating that the RRTs of the 25 components are comparatively stable. A total of 6, 4, and 5 peaks were found in *Mahuang*, *Fuzi*, and *Xixin*, respectively, and eight peaks were found in the three herbs. Nine compounds were detected based on the reference standards shown in Table 2. Peaks 2–6 derived from *Mahuang* were identified as norephedrine, norpseudoephedrine, ephedrine, pseudoephedrine, and methylephedrine, respectively. Peaks 10–12 derived from *Fuzi* were identified as benzoylmesaconine, benzoylaconine, and benzoylhypaconine, respectively. Peak 13 derived from *Xixin* was identified as 3,4,5-trimethoxytoluene.

### 3.2. MFXD Relieved Nasal Symptoms in a Rat Model of AR

**Figure 3:**
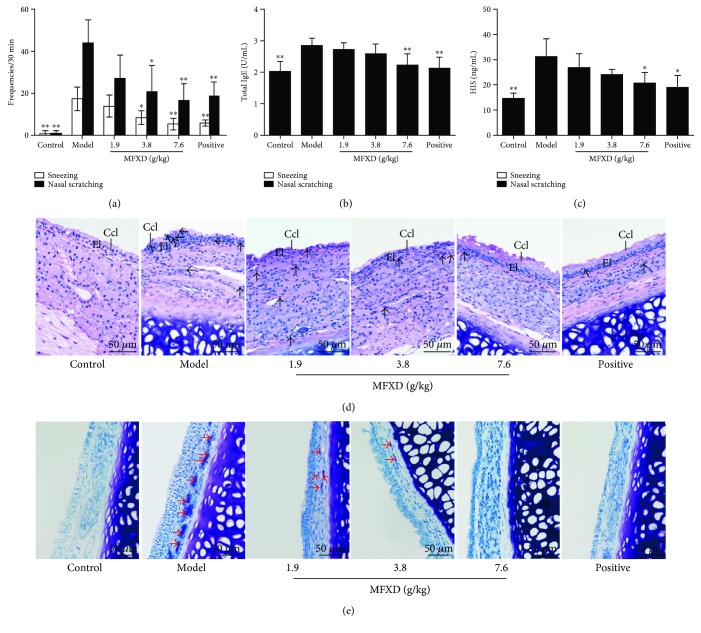
Effect of MFXD on OVA-induced allergic rhinitis in rat. (a) The frequencies of sneezing and nasal scratching of rats were counted for 30 min immediately after the last challenge by nasal instillation with OVA solution on day 31. Blood samples were collected and the serums were obtained by centrifugation. The total IgE (b) and HIS (c) levels in serum were detected by ELISA. The nasal mucosa samples were collected and stained with (d) hematoxylin and eosin (HE) and (e) toluidine blue (TB). Epithelial layer (El), ciliated cell layer (Ccl), leucocytes (represented by black arrows), and mast cells (represented by red arrows) were marked on the images. Data are expressed as mean ± SD; *N* = 8 rats; ^∗^*p* < 0.05, ^∗∗^*p* < 0.01 versus model.

The frequencies of sneezing and nasal scratching in rats were counted for 30 min to evaluate the therapeutic effect of MFXD after the last nasal challenge with OVA solution. As shown in Figure 3(a), the frequencies of sneezing and nasal scratching of rats in the AR model group significantly increased with an average of 17.38 and 44.00 times, respectively, compared with those in the control group (*p* < 0.01), wherein the rats did not show obvious sneezing and nasal scratching. After oral administration of MFXD, the nasal symptoms in rats of AR were relieved evidently. The frequencies of sneezing and nasal scratching in MFXD (3.8 g/kg) and MFXD (7.6 g/kg) groups significantly decreased compared with those in the AR model group (*p* < 0.05 and *p* < 0.01, resp.). The frequencies of sneezing and nasal scratching in the MFXD (7.6 g/kg) group were 5.38 and 16.63, respectively, demonstrating the effective therapeutic effect of MFXD on the nasal symptoms in an AR rat model and that such effect is as good as that in the positive group.

### 3.3. MFXD Reduced the Serum Levels of Total IgE and HIS in a Rat Model of AR

AR is a type I allergic disease induced by IgE-mediated inflammation and characterized by the release of HIS [30]. As summarized in Figure 3(b), the total IgE levels in rats in MFXD (7.6 g/kg) and positive groups were 2.23 and 2.13 U/mL, respectively, which were significantly reduced compared with that of 2.86 U/mL in the AR model group (*p* < 0.01; *p* < 0.01). In addition, the concentrations of serum HIS in the high-dose MFXD group and positive group were 20.58 and 19.07 ng/mL, respectively, which were also significantly reduced compared with that in the AR model group (*p* < 0.05; *p* < 0.05).

### 3.4. MFXD Relieved the Histopathological Injuries in the Nasal Mucosa in a Rat Model of AR

HE and TB staining examinations were conducted to evaluate the effect of MFXD on the histopathological changes in the nasal mucosa. The HE (Figure 3(d)) and TB (Figure 3(e)) staining results show that rats in the control group displayed no obvious visible nasal lesions. However, nasal respiratory epithelium disruption, leucocyte and mast cell infiltration, and ciliated cell reduction were observed in the nasal mucosa sections of AR model rats. By contrast, compared with the model group, the groups administered with MFXD and positive agent, especially in MFXD (7.6 g/kg) and positive groups, showed significantly alleviated nasal mucosa injuries caused by AR.

### 3.5. MFXD Reduced the Serum Levels of Th1 and Th2 Cytokines (IFN-*γ* and IL-4)

**Figure 4:**
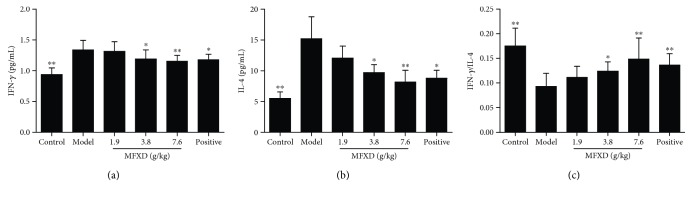
Effect of MFXD on serum IFN-*γ* and IL-4 in OVA-induced rat of allergic rhinitis. Blood samples were collected after the last nasal challenge and the serums were obtained by centrifugation. The IFN-*γ* (a) and IL-4 (b) levels in serum were detected by ELISA. (c) The IFN-*γ*/IL-4 values in all groups were calculated. Data are expressed as mean ± SD; *N* = 8 rats; ^∗^*p* < 0.05, ^∗∗^*p* < 0.01 versus model.

IFN-*γ* and IL-4 are the immune cytokines mainly released by Th1 and Th2 cells, respectively [9]. IFN-*γ* and IL-4 measurements were performed to indirectly indicate the status of Th1 and Th2 responses. As depicted in Figure 4, the serum levels of IFN-*γ* and IL-4 in AR model rats significantly increased compared with those in the control group. After oral administration of MFXD and loratadine, the serum levels of IFN-*γ* and IL-4 significantly decreased (except in the low-dose MFXD group), especially in MFXD (7.6 g/kg) rats (*p* < 0.01), compared with those in the model group. The serum IFN-*γ* levels in control, AR model, and MFXD (7.6 g/kg) groups were 0.94, 1.34, and 1.15 pg/mL, respectively, and the corresponding serum IL-4 levels of these groups were 5.52, 15.21, and 8.19 pg/mL, respectively. To evaluate the status of Th1/Th2 balance, we calculated the IFN-*γ*/IL-4 values in all groups (Figure 4). Compared with the IFN-*γ*/IL-4 value of 0.17 in the control group, that in the AR model group significantly decreased to 0.09 (*p* < 0.01). In addition, the IFN-*γ*/IL-4 values in MFXD (3.9 g/kg), MFXD (7.6 g/kg), and positive groups significantly increased (*p* < 0.05, *p* < 0.01, and *p* < 0.01, resp.) compared to that in the model group.

### 3.6. Effect of MFXD on the Percentages of CD3^+^CD4^+^IFN-*γ*^+^ Th1 and CD3^+^CD4^+^IL-4^+^ Th2 Cells in Peripheral Blood

**Figure 5:**
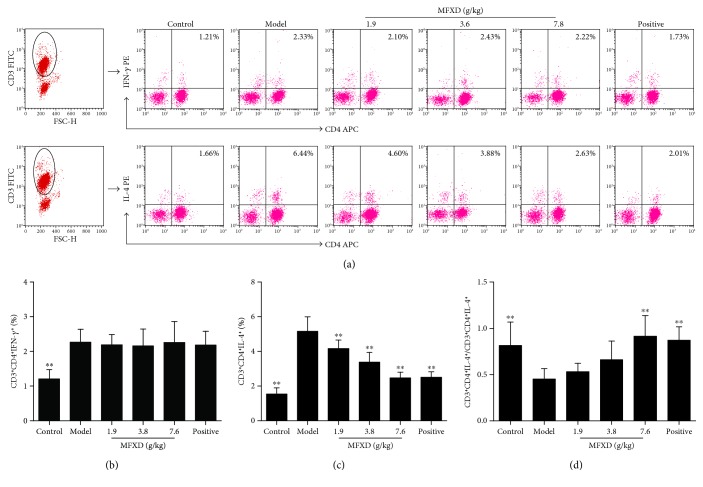
Effect of MFXD on the percentages of CD3^+^CD4^+^IFN-*γ*^+^ Th1 and CD3^+^CD4^+^IL-4^+^ Th2 cells in peripheral blood mononuclear cells (PBMCs) of AR rats. PBMCs of rats were separated from anticoagulant blood sample, stained with fluorescently labeled anti-rat antibodies, and analyzed by flow cytometry. (a) Representative flow cytometry dot plots for each groups, and the plots in the upper right quadrant indicate the percentage of CD3^+^CD4^+^IFN-*γ*^+^ Th1 and CD3^+^CD4^+^IL-4^+^ Th2 cells among PBMCs. Percentages of (b) CD3^+^CD4^+^IFN-*γ*^+^ Th1 cells and (c) CD3^+^CD4^+^IL-4^+^ Th2 cells in each groups. (d) Ratios of CD3^+^CD4^+^IFN-*γ*^+^ Th1 and CD3^+^CD4^+^IL-4^+^ Th2 cells were calculated. Data are expressed as mean ± SD; *N* = 6 rats; ^∗∗^*p* < 0.01 versus model.

Th1 and Th2 cells differentiated from CD3^+^CD4^+^ lymphocytes and the percentages of CD3^+^CD4^+^IFN-*γ*^+^ Th1 and CD3^+^CD4^+^IL-4^+^ Th2 cells were analyzed and measured in PBMCs via flow cytometry. As shown in Figures 5(a) and 5(b), the percentage of CD3^+^CD4^+^IFN-*γ*^+^ Th1 cells was significantly higher in the AR model group than in the normal control group (*p* < 0.01), whereas no obvious changes were observed among the model, MFXD, and positive groups. As shown in Figures 5(a) and 5(c), the percentage of CD3^+^CD4^+^IL-4^+^ Th2 cells in the AR model group was 5.16%, significantly higher than the 1.55% in the normal control group (*p* < 0.01). However, the percentage of CD3^+^CD4^+^IL-4^+^ Th2 cells significantly decreased after administration with MFXD and positive reagent (*p* < 0.01), and the percentages in MFXD (7.6 g/kg) and positive groups were 2.47% and 2.53%, respectively. Moreover, the ratio of CD3^+^CD4^+^IFN-*γ*^+^ Th1 and CD3^+^CD4^+^IL-4^+^ Th2 cells was calculated (Figure 5(d)), and the ratios in MFXD (7.6 g/kg) and positive groups significantly increased compared with that in the AR model group (*p* < 0.01) but did not significantly differ from that in the control group (*p* > 0.05).

### 3.7. Effect of MFXD on mRNA Expression Levels of IFN-*γ*, IL-4, T-bet, GATA-3, STAT-1, and STAT-6 in the Nasal Mucosa

**Figure 6:**
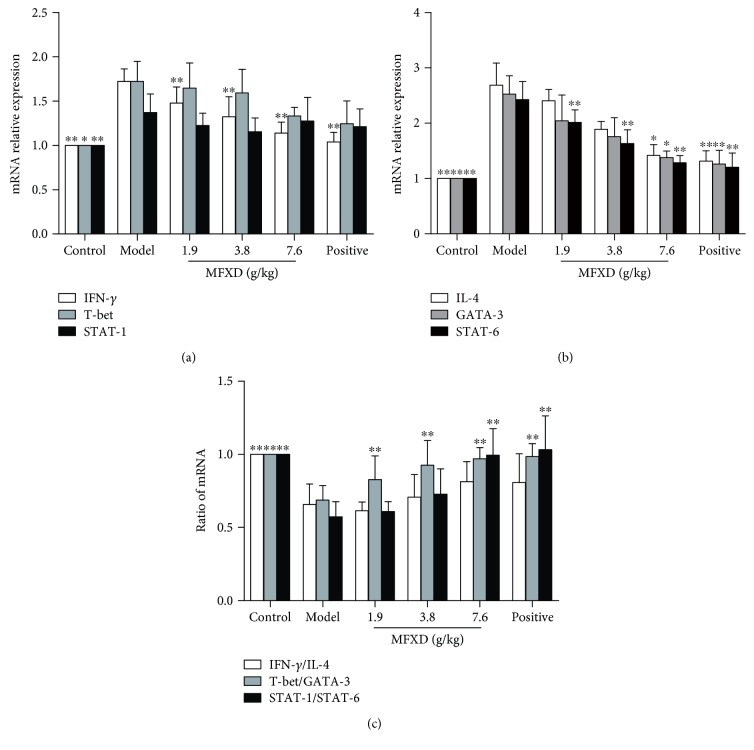
Effect of MFXD on the mRNA expression of IFN-*γ*, IL-4, T-bet, GATA-3, STAT-1, and STAT-6 in the nasal mucosa. Nasal mucosa samples from rats in different groups were obtained after the last nasal challenge, and total RNA was isolated and analyzed with qRT-PCR. (a) The mRNA expression of IFN-*γ*, T-bet, and STAT-1 in the nasal mucosa. (b) The mRNA expression of IL-4, GATA-3, and STAT-6 in the nasal mucosa. (c) The ratios of IFN-*γ*/IL-4, T-bet/GATA-3, and STAT-1/STAT-6 are shown. Data are expressed as mean ± SD; *N* = 6 rats; ^∗^*p* < 0.05, ^∗∗^*p* < 0.01 versus model.

T-bet and GATA-3 are important transcription factors that directly or indirectly regulate the differentiation and development of Th1 and Th2 cells [31]. IFN-*γ* activates the T-bet gene and induces the expression of T-bet through the Janus kinase/signal transducer and activator of transcription 1 signal transduction pathway [32, 33]. In addition, IL-4 increases the expression of GATA-3 by activating signal transducer and activator of transcription 6 (STAT-6) and then improves the development of Th2 cells [34]. As shown in Figures 6(a) and 6(b), the mRNA expression levels of IFN-*γ*, IL-4, T-bet, GATA-3, STAT-1, and STAT-6 in the nasal mucosa were significantly higher in the AR model group than in the control group. After intragastric administration of MFXD and positive drug, the expression levels of IFN-*γ* and STAT-6 in all groups significantly decreased and the expression levels of IL-4 and GATA-3 in MFXD (7.6 g/kg) and positive groups significantly decreased compared with those in the AR model group.  Figure 6(c) shows the IFN-*γ*/IL-4, T-bet/GATA-3, and STAT-1/STAT-6 values, which were calculated to evaluate Th1 and Th2 responses. IFN-*γ*/IL-4, T-bet/GATA-3, and STAT-1/STAT-6 values in the AR model group significantly decreased compared with those in the control group, and T-bet/GATA-3 ratios were significantly higher in MFXD and positive groups than that in the model group. Moreover, compared with that in the AR model group, the STAT-1/STAT-6 values in MFXD (7.6 g/kg) and positive groups significantly increased and the IFN-*γ*/IL-4 values in administered groups also tended to increase.

## 4. Discussion

AR is one of the most common allergic diseases that do not only adversely affect patients' quality of life but also induce multiple relevant complications [4, 5]. Researchers have successfully established different animal models of AR by using OVA as an allergen and Al(OH)_3_ as an adjuvant [35, 36]. In this study, a rat model of AR was successfully established through intraperitoneal injection of OVA and Al(OH)_3_ suspension and nasal instillation with 5% OVA solution. In the AR model group, the rats displayed extremely frequent sneezing and nasal scratching and significantly increased serum levels of total IgE and HIS; moreover, obvious epithelial disruption, leucocyte and mast cell infiltration, and cilia cell reduction were observed in nasal mucosa sections; these results indicated that the rat model of AR was successfully established and presented evident AR responses.

MFXD is a classical Chinese traditional formula that has been widely used in clinics to treat AR in Asian countries [20, 21]. In this study, three doses of MFXD were orally administrated to treat AR in rats. The frequencies of sneezing and nasal scratching in rats significantly decreased (except those in the low-dose MFXD group), and the serum levels of total IgE and HIS under high-dose MFXD treatment significantly decreased relative to those in the model group. In addition, MFXD significantly alleviated the nasal mucosa injuries caused by AR. Therefore, MFXD obviously exerted therapeutic effect on a rat model of AR, especially under treatment with high dose of MFXD.

AR is a Th2-polarized allergic disease, and imbalance of Th1/Th2 cells is speculated to be an important factor that results in AR [8–11]. Differentiation from Th0 cell to Th2 cell is obviously enhanced in AR; as a result, the release of Th2 cytokines is increased, accelerating the expression of transcription factors and signal transducers and activators of transcription, such as GATA-3 and STAT6, which play important roles in Th2 immune response [31–33]. In addition, excessive Th2 cytokine inhibits the differentiation of Th0 cell into Th1 cell, reducing the production of factors related to Th1 immune response, such as IFN-*γ*, T-bet, and SATA-1, leading to the imbalance in Th1/Th2 cells [34]. Studies have reported that in animal models induced by OVA, Th1 response is inhibited [37, 38] or is not obviously affected [39, 40], whereas Th2 response is enhanced. However, the serum IFN-*γ* content, the percentage of CD3^+^CD4^+^IFN-*γ*^+^ Th1 cells in PBMCs, and the mRNA expression levels of IFN-*γ*, T-bet, and SATA-1 were all significantly increased compared with those in the normal control group; the same trend was observed for the serum IL-4 content, the percentage of CD3^+^CD4^+^IL-4^+^ Th2 cells in PBMCs, and the mRNA expression levels of IL-4, GATA-3, and SATA-6. In this study, Th1 and Th2 responses were simultaneously enhanced in a rat model of AR, consistent with the results of Kim et al. who also used a mouse model of AR [41]. Two reasons are speculated to explain this phenomenon. First, although IgE-mediated AR is considered a disease primarily mediated by Th2, allergen-specific Th1 also plays an important role in allergic inflammation [42], especially in chronic allergic diseases, such as atopic dermatitis and chronic allergic asthma; moreover, the Th1 cytokine IFN-*γ* promotes allergen penetration through the respiratory epithelium and aggravates allergic inflammation [43, 44]. Second, Th1 response possibly played different roles in different periods after AR has developed. As reported, the serum levels of the Th1 cytokine IFN-*γ* in a rat model of OVA-induced AR obviously increased within 24 h after induction of AR and then decreased [45].

Although Th1 and Th2 responses were enhanced simultaneously in a rat model of AR, the imbalance in Th1/Th2 cells significantly changed. Compared with those in the control group, the following ratios significantly decreased in the AR model group: ratios of serum IFN-*γ*/IL-4; CD3^+^CD4^+^IFN-*γ*^+^ Th1 (%)/CD3^+^CD4^+^IFN-*γ*^+^ Th1 (%) in PBMCs; and mRNA expression of IFN-*γ*/IL-4, T-bet/GATA-3, and STAT-1/STAT-6. After oral administration of MFXD, all of the values tended to increase and high dose of MFXD significantly enhanced the values, consistent with the result for the positive reagent, indicating that MFXD can regulate the AR-induced imbalance in Th1/Th2 cells.

In conclusion, MFXD exerts a good therapeutic effect against OVA-induced allergic inflammation in a rat model of AR as seen in the reduced frequencies of sneezing and nasal scratching in rats and in the reduced serum levels of total IgE and HIS. MFXD regulates the AR-induced imbalance in Th1/Th2 cells by attenuating Th1 and Th2 responses simultaneously, such as reducing the serum levels of IFN-*γ* and IL-4 and mRNA expression levels of IFN-*γ*, IL-4, GATA-3, and STAT-6. This study provides some valuable information on the immunoregulatory effect of MFXD on the treatment of AR in future clinical studies.

## Figures and Tables

**Table 1:** Primer sequences for quantitative real-time PCR.

Gene	Primer	Sequences (5′–3′)
IFN-*γ*	Forward	CTGCTGATGGGAGGAGATGT
	Reverse	TTTGTCATTCGGGTGTAGTCA
IL-4	Forward	GAGACTCTTTCGGGCTTTTCG
	Reverse	CAGGAAGTCTTTCAGTGATGTGG
T-bet	Forward	CAACAACCCCTTTGCCAAAG
	Reverse	TCC CCCAAGCAGTTGACAGT
GATA-3	Forward	CTCGGCCATTCGTACATGGAA
	Reverse	GGATACCTCTGCACCGTAGC
STAT-1	Forward	TGACGAGGTGTCTCGGATAGT
	Reverse	GTAGCAGGAGGGAATCACAGA
STAT-6	Forward	CTGCCAAAGACCTGTCCATT
	Reverse	GGTAGGCATCTGGAGCTCTG
*β*-Actin	Forward	ACCAACTGGGACGACATGGAGAA
	Reverse	GTGGTGGTGAAGCTGTAGCC

**Table 2:** Identification of compounds of MFXD by UPLC-MS/MS.

Number	RRT/min^∗^	Compound	Positive ion (*m*/*z*)	Elemental composition	Chemical structures	Source
1	5.67	Norephedrine	152.1	C_9_H_13_NO	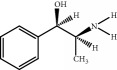	^a^
2	6.13	Norpseudoephedrine	152.1	C_9_H_13_NO	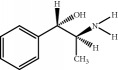	^a^
3	6.99	Ephedrine	166.1	C_10_H_15_NO	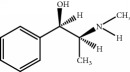	^a^
4	7.49	Pseudoephedrine	166.1	C_10_H_15_NO	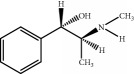	^a^
5	7.97	Methylephedrine	180.1	C_11_H_17_NO	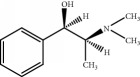	^a^
6	17.95	Benzoylmesaconine	590.3	C_31_H_43_NO_10_	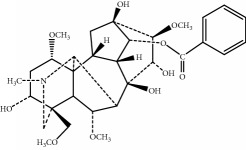	^b^
7	19.17	Benzoylaconine	604.3	C_32_H_45_NO_10_	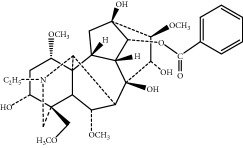	^b^
8	20.10	Benzoylhypaconine	574.3	C_31_H_43_NO_9_	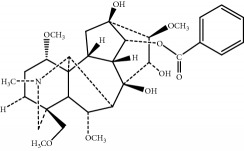	^b^
9	23.47	3,4,5-Trimethoxytoluene	183.1	C_10_H_14_O_3_	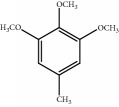	^c^

^∗^RRT: relative retention time. ^a^*Ephedrae*; ^b^*Radix Aconiti Lateralis*; ^c^*Asarum.*
